# The Experience of International Medical Graduates (IMGs) at Birmingham and Solihull Mental Health Foundation Trust (BSMHFT): A Quality Improvement Project

**DOI:** 10.1192/bjo.2024.436

**Published:** 2024-08-01

**Authors:** Shahnila Sohail, Tabassum Mirza, Muhammad Abiola, Anum Asim, Adedamola Adeyanju

**Affiliations:** Birmingham and Solihull Mental Health Foundation Trust, Birmingham, United Kingdom

## Abstract

**Aims:**

To improve the overall experience of IMG Doctors at BSMHFT. To demonstrate this, we targeted an increase in percentage of doctors rating their experience as excellent in our survey.

**Methods:**

Employing the “Model for improvement”, we co -produced all aspects of project with subject matter experts (IMGs).

- What are we trying to accomplish? We co-produced process map/aim.
- How will we know that a change is an improvement? Co-produced survey, circulated monthly, data collected and analysed.
- What change can we make that will result in an improvement: Co-produced change ideas from process map, survey data and weekly meetings. Commenced testing some change ideas in this phase.
  Other QI tools utilized include Driver diagram and family of measures.
- Change strategies (PDSA cycles):
  These include:

- IMG specific session at Trust Induction. 15 minutes slot allocated to introduce project and encourage involvement.
- IMG whatsapp group.
- IMG Forum.
- Dedicated Email Inbox.
- Learning/career progression sessions.
- Social events.
- IMG representative.

Following organised sessions number of attendees recorded and feedback obtained via survey.

**Results:**

Outcome measure (Aim) - 5 months of survey data obtained. An average of 10 IMGs responded to monthly survey. Data presented on statistical process chart (SPC) revealed a median value of 33% of respondents (IMGs) rated their experience as excellent.

**Process measures** –

- *Trust Induction* – 2 PDSA cycles completed. 15 IMGS joined the Whatsapp group following induction sessions. 24 IMGs joined the IMG mailing list.
- *Whatsapp group* – Completed 9 PDSA cycles. Average of 3 IMGs joined per week. 69 members at present. Data indicates informal posts, planned activities, information sharing, and spontaneous queries encouraged engagement.
- *IMG Forum* – one PDSA cycle completed. 20 IMGs attended. Feedback was obtained and 63% of respondents rated the effectiveness of the session as excellent.
- *Social event* – one event arranged; 16 IMGs attended.

**Conclusion:**

From this phase of the QI project, we have been able to foster an increased sense of community peer support and camaraderie amongst IMGs. This is highlighted by increased numbers in WhatsApp group, mailing list and attendance at events.

The change ideas positively impacted participation, engagement, and satisfaction with the project providing a previously unavailable psychological safe space.

33% of respondents rated their overall experience as excellent from the monthly surveys that were sent out.

In terms of next steps, we aim to implement other change ideas and aim to increasing respondents’ rating as excellent to 50% by December 2024.

